# The Medical Student and His Preceptor

**Published:** 1891-03

**Authors:** 


### Editorial.

### THE MEDICAL STUDENT AND HIS PRECEPTOR.

To anyone who is not cognizant of the facts in the case, the attitude of many medical schools towards aspirants to a position in the ranks of the profession, is, to say the least, surprising; and to one who is disposed to regard the profession as a high calling, very humiliating. The multiplicity of medical colleges, and the intense desire to have as large a number of matriculants as possible, has to a large extent entirely run away with the ideas of many who are managing these institutions, and as a result, we find many colleges, whose age and former position would lead us to hope that they might do better, bidding and buying in the market in a manner that would do justice to a very sharp trader in the marts of business.

It has long since been proven that the reforming of these practices is to a large extent beyond hope, but we would respectfully call attention to some of the facts in the case, in the hope that there may be some good done by touching those who have to a certain extent the guidance of the young men who are entering upon the study of medicine, viz: the medical men who act as preceptors for them.

In this age there is the one absorbing thought of cheapness, not considering the facts that colleges, as well as other things in this world cannot be valuable and cheap at the same time. In the South it is a fact that the regular fees charged are far too cheap any way, and when we reflect that there are many enterprising schools that are offering freely to take students at the rate of twenty-five dollars per head, we see that the demands for additional reductions are imperative. Why, we would ask, should any school be asked to donate to a young man the amount of fifty dollars or more, simply because he wants to study medicine? Surely, if he has not among his friends and relatives, some one who can assist him to this extent, he has not the right to demand it of an institution that has no interest in him, and whose instruction has as much money value as the professional work of its instructors commands. And yet the medical schools, just as if the time of the teachers were worth nothing, are offering to the "deserving young men of limited means" without money and without price, their unparallelled clinical facilities, and all the other trimmings of a first class medical course. Letters are sent broadcast, in which "we are informed by a mutual friend that you contemplate taking a course of lectures," and urging upon him to get a proposition from them before going elsewhere. So widespread is this state of affairs that we are sure that this cap will fit in many cases. Why, we ask, should the preceptor of a young man permit him to be led away by these offers of cutting rates, any more than he would advise a man to consult a physician who offers to make war upon the usual charges in his community? We believe that it is largely due to thoughtlessness, and if he would consider the matter he would not advise any student to accept a reduction under such circumstances. Besides, almost every state has arranged an avenue for those who are totally unable to pay; in the State of Georgia alone thirty young men are entitled to free medical tuition.

Again, many students are induced to seek a certain school because of a well known low standard, and any college that has the conscience to reject an applicant occasionally is avoided on account of the uncertainty of obtaining a degree. This seems to be peculiarly hard upon those institutions that are trying to meet in some degree the demands for a higher standard of education. We know of an institution whose age, and the names of its faculty, should lead us to expect that some attention would be paid to keeping up a standard, in which there has been one rejection of a student upon final examination in four years, in which time counting less than two hundred have been graduated. Well might one of a graduating class of almost one hundred have remarked (as he did) that he "supposed that he would graduate, as no one had ever been rejected." Such a state of affairs is truly lamentable, and in the selection of such a school the preceptor should warn the student, as he is not a competent judge of such matters, and is the more apt to be misled. The student should be taught that it is not merely the obtaining of a degree that he wants, but a sufficient basis upon which to begin his work, and this can only be obtained by patient study, and not by simply spending two sessions at a medical school, where this means a guarantee of graduation. That there are such medical colleges is a fact that ample evidence will bear out.

Again we would say that the safeguard of the student of medicine is the intelligent advice of his preceptor, and with him it should be not a matter of cheapness, or of the guarantee of a diploma, but the school that will give the most instruction.

